# A systematic review and meta-analysis of outcomes associated with development of surgical site infection after lower-limb revascularization surgery

**DOI:** 10.1177/17085381241290039

**Published:** 2024-10-03

**Authors:** Aidan M Kirkham, Jasmine Candeliere, Sudhir K Nagpal, Henry T Stelfox, Dalibor Kubelik, George Hajjar, Derek R MacFadden, Daniel I McIsaac, Derek J Roberts

**Affiliations:** 1 Division of Vascular and Endovascular Surgery, Department of Surgery, 6363 University of Ottawa, Ottawa, ON, Canada; 2 Clinical Epidemiology Program, The Ottawa Hospital Research Institute, The Ottawa Hospital, Ottawa, ON, Canada; 3 School of Epidemiology & Public Health, Faculty of Medicine, 6363 University of Ottawa, Ottawa, ON, Canada; 4 Department of Critical Care Medicine, Medicine, and Community Health Sciences, University of Calgary, Calgary, AB, Canada; 5 Division of Infectious Disease, Department of Medicine, 6363 University of Ottawa, Ottawa, ON, Canada; 6 Departments of Anesthesiology and Pain Medicine, University of Ottawa and The Ottawa Hospital, Ottawa, ON, Canada; 7 Institute for Clinical Evaluative Sciences, Toronto, ON, Canada

**Keywords:** Lower-limb revascularization surgery, surgical site infection, clinical outcomes, systematic review, meta-analysis

## Abstract

**Objectives:**

Although surgical site infection (SSI) is a commonly used quality metric after lower-limb revascularization surgery, outcomes associated with development of this complication are poorly characterized. We conducted a systematic review and meta-analysis of studies reporting associations between development of an SSI after these procedures and clinical outcomes and healthcare resource use.

**Methods:**

We searched MEDLINE, Embase, CENTRAL, and Evidence-Based Medicine Reviews (inception to April 4th, 2023) for studies examining adjusted associations between development of an SSI after lower-limb revascularization surgery and clinical outcomes and healthcare resource use. Two investigators independently screened abstracts and full-text citations, extracted data, and assessed risk of bias. Data were pooled using random-effects models. Heterogeneity was assessed using I^2^ statistics. GRADE was used to assess estimate certainty.

**Results:**

Among 6671 citations identified, we included 11 studies (n = 61,628 total patients) that reported adjusted-associations between development of an SSI and 13 different outcomes. Developing an SSI was associated with an increased adjusted-risk of hospital readmission (pooled adjusted-risk ratio (aRR) = 3.55; 95% CI (confidence interval) = 1.40–8.97; n = 4 studies; n = 13,532 patients; I^2^ = 99.0%; moderate certainty), bypass graft thrombosis within 30-days (pooled aRR = 2.09; 95% CI = 1.41–3.09; n = 2 studies; n = 23,240 patients; I^2^ = 51.1%; low certainty), reoperation (pooled aRR = 2.69; 95% CI = 2.67–2.72; n = 2 studies; n = 23,240 patients; I^2^ = 0.0%; moderate certainty), bleeding requiring a transfusion or secondary procedure (aRR = 1.40; 95% CI = 1.26–1.55; n = 1 study; n = 10,910 patients; low certainty), myocardial infarction or stroke (aRR = 1.21; 95% CI = 1.02–1.43; n = 1 study; n = 10,910 patients; low certainty), and major (i.e., above-ankle) amputation (pooled aRR = 1.93; 95% CI = 1.26–2.95; n = 4 studies; n = 32,859 patients; I^2^ = 83.0; low certainty). Development of an SSI >30-days after the index operation (aRR = 2.20; 95% CI = 1.16–4.17; n = 3 studies; n = 21,949 patients; low certainty) and prosthetic graft infection (aRR = 6.72; 95% CI = 3.21–12.70; n = 1 study; n = 272 patients; low certainty) were both associated with an increased adjusted-risk of major amputation. Prosthetic graft infection was also associated with an increased adjusted-risk of mortality >30-days after the index procedure (aRR = 6.40; 95% CI = 3.32–12.36; n = 1 study; n = 272 patients; low certainty).

**Conclusions:**

This systematic review and meta-analysis suggests that development of an SSI after lower-limb revascularization surgery significantly increases patient morbidity and healthcare resource use. SSI is therefore a valuable quality metric after these surgeries. However, current estimates are based on heterogenous, low-to-moderate certainty evidence and should be confirmed by large, multicenter, cohort studies.

## Introduction

Surgical site infection (SSI) is a common, potentially preventable complication after lower-limb revascularization surgery (i.e., endarterectomy, patch angioplasty, or arterial bypass) thought to be associated with an increased risk of healthcare resource use, morbidity, and potentially even limb loss and mortality.^1–3^ SSI has been suggested to be “the most important complication” of open vascular surgery, and rates of this complication are often used as a quality indicator by healthcare systems.^4^ Systematic reviews and meta-analyses have reported that the incidence of an SSI is approximately 6% after all open infrainguinal revascularization procedures^5^ and 8% after surgeries utilizing a groin incision.^6^ Further, the cumulative incidence of prosthetic graft infection (the most severe type of SSI) after infrainguinal bypass is approximately 0.4%.^5^

Despite this, the relationship between SSI and outcomes after lower-limb revascularization surgery (and therefore the value of using SSI as a quality metric) remains poorly characterized. Although several studies have examined these relationships,^7–17^ interpretation of their results is limited by inter-study heterogeneity. Available studies often included patients who had varying comorbidities, different indications for lower-limb revascularization surgery, and who underwent different types of surgery.^7,10,12,13^ They also frequently examined different outcomes,^9,11,14^ used heterogenous outcome definitions or different effect measures,^8,9,16^ measured outcomes at varying timepoints,^13,16,17^ and adjusted their effect measures for different confounding variables.^9,11,16^ These differences may explain why some studies report conflicting SSI-outcome relationships.^8,10,16,17^ There is also limited information on outcomes associated with development of a prosthetic patch or bypass graft infection.^12^

We conducted a systematic review and meta-analysis to synthesize and evaluate adjusted-associations between development of an SSI and clinical outcomes and healthcare resource use in adults after lower-limb revascularization surgery. The study purpose was to determine the evidence supporting use of SSI as a quality metric after these procedures. We also sought to explore research gaps and heterogeneity in the published literature on SSI after lower-limb revascularization surgery, including whether SSI-outcome associations vary based on study risks of bias, patient and operative characteristics, study setting, time of SSI development, and SSI severity.

## Methods

### Protocol, registration, reporting, and ethics

We pre-specified our methods according to the Preferred Reporting Items in Systematic Reviews and Meta-Analyses-Protocols (PRISMA-P) statement^18^ (Supplemental Table 1). The study followed recommendations for conducting systematic reviews and meta-analyses of prognostic factor studies.^19^ The protocol was registered on the Prospective International Register of Systematic Reviews (PROSPERO) before study start (Registration Number: CRD42022329543). Systematic reviews are exempt from ethics review at our institution.

### Eligibility criteria

We included studies that: 1) included adults (≥18-years) who underwent lower-limb revascularization surgery; 2) reported adjusted-odds ratios (aORs), -relative risks (aRRs), or -hazard ratios (aHRs) (and surrounding 95% confidence intervals [CIs]) describing associations between development of an SSI (or a distinct severity of SSI) and clinical outcomes and healthcare resource use. We considered lower-limb revascularization surgery to include iliofemoral/femoral endarterectomy and/or patch angioplasty and aortofemoral, iliofemoral, femoral-popliteal, femoral-tibial/peroneal, axillofemoral, and femoral-femoral bypass.^20^ We excluded studies that did not account for confounding (e.g., through multivariable adjustment)^21^ or exclusively examined high-risk cohorts (e.g., only those with diabetes).

### Information sources and search strategy

We searched MEDLINE, Embase, Evidence-Based Medicine Reviews (ACP Journal Club; Database of Systematic Reviews; Methodology Register Database; Database of Abstracts of Reviews of Effects; Health Technology Assessment Database; and National Health Service Economic Evaluation Database), and the Cochrane Central Register of Controlled Trials (inception date to April 4th, 2023), without restrictions. The initial search strategy was created in consultation with an information-scientist/medical librarian and included Medical Subject Heading (MeSH)/Emtree terms and keywords covering the themes *lower-limb revascularization surgery* and *infection*. The initial strategy was piloted and refined during iterative pilot searches by the information scientist. The penultimate search strategy was peer-reviewed by a second, independent information-scientist as per the Peer Review of Electronic Search Strategies (PRESS) guidelines^22^ (see Supplemental Table 2 for full electronic search strategy). We also used the PubMed “related articles” feature and searched bibliographies of included studies and relevant review articles identified during searches.

### Selection process

Two investigators (A.M.K. and J.C.) independently reviewed the titles and abstracts of citations identified by the search using Rayyan (https://www.rayyan.ai/).^23^ These investigators then independently reviewed the full-texts of all potentially relevant citations to determine final eligibility. Inter-investigator agreement on full-text inclusion was assessed using the kappa (κ) statistic.^24^ Disagreements regarding full-text article inclusion were resolved by consensus.

### Data collection and risk of bias assessment

Two investigators (A.M.K. and J.C.) independently extracted data using a pre-designed data extraction spreadsheet piloted on three included studies. We extracted the following data: 1) study design, data source, and setting; 2) patient recruitment and follow-up period; 3) patient and procedural characteristics, including the proportion of different revascularization and urgent/emergent procedures; 4) adjusted-associations between SSI (or different severities of SSI) and clinical outcomes or healthcare resource use; and 5) confounding factors adjusted for in multivariable analyses. Risk of bias was independently evaluated by two investigators (A.M.K and J.C.) using the Quality in Prognosis Studies (QUIPS) tool.^25^ Disagreements regarding data collection and risk of bias assessments were resolved by consensus.

### Outcomes

The primary outcome was the adjusted-association between development of an SSI and clinical outcomes or healthcare resource use (as defined and reported by study authors). Secondary outcomes were adjusted-associations between different severities of SSI (as defined by the Centers for Disease Control (CDC),^26^ Szilagyi,^27^ or other SSI classification systems used by study authors) and early (in-hospital/≤30-days) and late (>30-days) SSI and clinical outcomes and healthcare resource use.

### Data synthesis and statistical analyses

#### Qualitative

We performed a narrative synthesis of included SSI-outcome relationships before considering meta-analyses.^28^ This involved tabulating the SSI type (e.g., any SSI or prosthetic graft SSI) and associated outcomes and outcome definitions, study data sources, and patient recruitment and follow-up periods. Two investigators (A.M.K. and D.J.R.) then used content analysis^29^ to inductively cluster the reported associated outcomes into four mutually exclusive categories created based on their content (graft-related outcomes, morbidities, mortality, or healthcare resource use).

#### Quantitative

Descriptive data were summarized using weighted means and standard deviations (SDs), medians and interquartile ranges (IQRs), and counts and percentages as appropriate. The primary measure used to describe adjusted SSI-outcome associations was aRRs as they are more readily interpretable than aORs and aHRs.^30^ As has been suggested in other studies, if the outcome incidence within the study population was <10%, we considered reported aORs and aHRs to approximate aRRs and reported them as aRRs.^31,32^ However, if the SSI incidence within the study population was ≥10%, we used validated methods to convert reported aORs and aHRs to aRRs,^33,34^ as has been done in other systematic reviews and meta-analyses.^35,36^ In studies where raw data describing counts or proportions of patients experiencing outcomes were not presented, we pooled the proportion of patients experiencing the outcome of interest in other reporting studies using DerSimonian and Laird random effects models^37^ to derive common proportions for effect measure transformation. For outcomes that only included studies reporting adjusted estimates without raw counts or proportions of patients experiencing reported outcomes, the square root of reported effect measures (aORs or aHRs) was used as an approximate conversion of these reported measures to aRRs as has been suggested.^34^

Adjusted associations with similar outcome definitions measured at similar time points were subsequently pooled using random-effects models.^37^ When estimates were calculated from the same data source and recruitment period across multiple studies, we included the estimate from the study with the largest sample size in the primary meta-analysis. In sensitivity meta-analyses, we recalculated summary estimates using a more adjusted estimate from a smaller study where this existed. We also preformed sensitivity analyses that excluded studies in which effect measures were converted using either optimal approximate^33^ or square-root transformations^34^ from our meta-analyses.

We calculated I^2^ statistics and conducted homogeneity tests to assess for inter-study heterogeneity.^38^ We considered I^2^ statistics >25%, >50%, and >75% to represent low, moderate, and high heterogeneity, respectively.^38^ In the presence of at least low inter-study heterogeneity, we conducted stratified meta-analyses and meta-regressions using random-effects models.^37^ We used the following predictors to explore inter-study heterogeneity: 1) high/moderate vs low risk of bias related to study participation and attrition, SSI and outcome description and measurement, or methods and reporting of statistical analyses; 2) whether associations were adjusted for a pre-specified minimum confounder set^39^ (age, sex, obesity, diabetes, chronic limb-threatening ischemia (CLTI), and groin incision) or above or below the median number of confounders used across all studies; 3) the proportion of patients undergoing aortofemoral bypass, axillofemoral bypass, a groin-only procedure, or an infrainguinal bypass; 5) the country or region of study origin; and 6) the proportion of patients with CLTI, who were female, underwent urgent/emergent surgery or were obese (defined as a body mass index (BMI) ≥30-kg/m^2^). The minimum confounder set^39^ was selected after a narrative review of studies examining predictors of outcomes after lower-limb revascularization surgery.^16,40–42^

We evaluated for small study effects potentially due to publication bias by inspecting funnel plots and conducting Egger’s test.^43^ Statistical analyses were performed using Stata SE version 17.0 (Stata Corporation, College Station, Texas, USA). p < 0.05 was considered significant for all analyses. Risk of bias graphics was generated using Review Manager version 5.4.

### Certainty assessment

Certainty in estimates was evaluated using Grading of Recommendations, Assessment, Development, and Evaluation (GRADE).^44^ This was done by first assessing the risk of bias, consistency, directness, precision, and risk of publication bias associated with each SSI-outcome estimate.^44^ The certainty of all estimates was subsequently defined as high (“further research is very unlikely to change our certainty in the estimate”), moderate (“further research is likely to have an important impact on our certainty in the estimate and may change the estimate”), or low (“further research is very likely to have an important impact on our certainty in the estimate and is likely to change the estimate”).^44^

## Results

### Search

**Figure 1.**
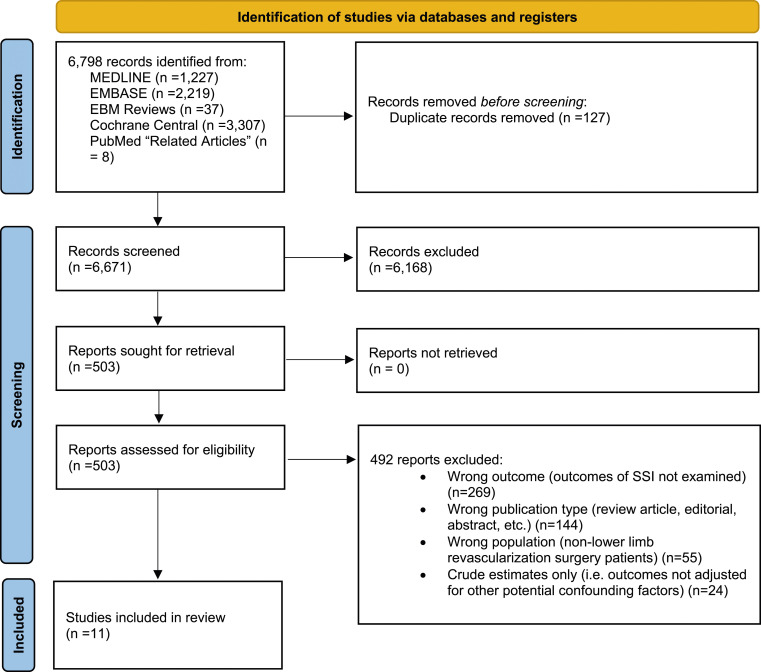
Flow of articles through the systematic review. MEDLINE, Embase, Evidence-Based Medicine Reviews, and the Cochrane Central Register of Controlled Trials Databases were searched from 1946 to April 4^th^, 2023.

Of 6,671 citations identified, we included 11 cohort studies^7–17^ (n = 61,628 patients) (see Figure 1 for the PRISMA flowchart). Inter-investigator agreement on full-text article inclusion was excellent (κ = 0.75; 95% CI = 0.59–0.90).

### Study characteristics

Characteristics of the 11 included studies^7–17^ are summarized in Table 1. Their median publication date was 2019 (IQR = 2016–2022). Most were prospective (63.6%), multicenter (54.5%) studies conducted in the United States (81.8%). These studies derived their data from the American College of Surgeons National Surgical Quality Improvement Program (ACS-NSQIP) (36.4%), institutional databases/registries (27.3%), the Society for Vascular Surgery Vascular Quality Initiative (SVS-VQI) (9.1%), the Vascular Study Group of New England (VSGNE) and SVS-VQI (9.1%), and other databases (18.2%). The median reported follow-up across studies was 30-days (IQR = 30–365-days). Studies most frequently defined SSIs according to the classification system proposed by the CDC^26^ (36.4%), study author definitions (18.2%), and the Szilagyi^27^ classification system (9.1%). Four^7,9,10,15^ studies did not report the system used to define SSIs. Most patients reportedly underwent elective (86.9%) infrainguinal bypass (99.1%) for CLTI (67.3%).

**Table 1.** Characteristics of the 11 studies included in the systematic review.

Study	Data source (country)	Prospective	SSI grading system	Centers	Recruitment period	No. of pts (% F^a^)	Surgery types (%)	Mean age, years	U/E surgery (%)	CLTI (%)	BMI, kg/m^2^	F/U, d
Kim *et al.* (2023)^7^	RPDR (USA)	No	NR	6	1995–2020	843 (40.0)	LEB = 100.0	71.0	NR	100.0	NR	30
Na *et al.* (2023)^8^	ACS-NSQIP (USA)	Yes	CDC^26^	NR	2014–2017	10910 (32.9)	FP = 56.8; FD = 30.4; PD = 9.9; Other = 2.0; FE = 0.9	66.9	NR	71.2	28.5	30
Pherson *et al.* (2022)^9^	SVS-VQI (USA)	No	NR	NR	2003–2017	20273 (31.8)	LEB = 100.0	66.0	17.2	67.5	BMI ≥30 kg/m^2^: 30.9%	365
Tsay *et al.* (2020)^10^	ACS-NSQIP (USA)	Yes	NR	>800	2012–2016	5304 (42.4)	FP = 100.0	Age ≥65: 62.0%	2.8	100.0	BMI ≥30 kg/m^2^: 28.4%	30
Bluemn *et al.* (2019)^11^	VSGNE and SVS-VQI (USA)	Yes	Szilagyi^27^	124	2008–2016	5842 (31.8)	LEB = 100.0	Age >70: 36.2%	23.8	56.8	28.0	30
Etkin *et al.* (2019)^12^	SI (USA)	No	AD	1	2014–2016	272 (41.2)	FP = 34.2; FD = 18.4; FE = 16.2; FF = 15.1; IoF = 7.7; AxF = 5.9; AoF = 2.6	73.4	NR	38.2	27.6	668
Farinas *et al.* (2017)^13^	SI (Spain)	Yes	CDC^26^	1	2008–2009	261 (11.9)	LEB = 81.2; AoF = 14.2; ^a^Other = 4.6	67.6	11.5	NR	BMI ≥30 kg/m^2^: 17.2%	1095
Aziz *et al.* (2016)^14^	ACS-NSQIP (USA)	Yes	CDC^26^	>250	2013	2646 (35.3)	FP = 58.9; FD = 28.6; PD = 8.7; ^b^Other = 2.5; FE = 1.3	67.7	5.7	70.9	BMI ≥30 kg/m^2^: 31.0%	30
McPhee *et al.* (2013)^15^	SI (USA)	Yes	NR	1	1995–2011	1543 (38.7)	LEB = 100.0	68.8	NR	84.5	NR	30
Greenblatt *et al.* (2011)^16^	ACS-NSQIP (USA)	Yes	CDC^26^	211	2005–2008	12330 (36.7)	FP = 42.5; FD = 25.2; FE = 14.4; FF = 10.9; PD = 7.1	67.4	7.5	46.0	28.1	30
Nguyen *et al.* (2007)^17^	PREVENT III (USA and Canada)	No	AD	83	2001–2003	1404 (36.1)	LEB = 100.0	69.0	NR	100.0	NR	365

Where ACS-NSQIP, American College of Surgeons National Surgical Quality Improvement Program; AD, author definition; AoF, aortofemoral bypass; AxF, axillofemoral bypass; BMI, body mass index; CDC, Centers for Disease Control; CLTI, chronic limb-threatening ischemia; CON, country; F, female; FE, femoral endarterectomy; FD, femoral-distal bypass; FF, femoral-femoral bypass; FP, femoral-popliteal bypass; F/U, follow-up; IoF; iliofemoral bypass; LEB, lower extremity bypass; NR, not reported; PD, popliteal-distal bypass; PREVENT III, PRoject or Ex-vivo vein graft ENgineering via Transfection III; Pts, patients; RPDR, Research Patient Data Registry; SI, single institution; SVS-VQI, Society for Vascular Surgery Vascular Quality Initiative; U/E, urgent/emergent; VSGNE, Vascular Study Group of New England.

^a^Patient sex breakdown was ascertained directly from the studies as reported by authors.

^b^Other includes profundoplasty and unspecified lower-limb arterial bypass.

### Risk of bias assessment

**Figure 2.**
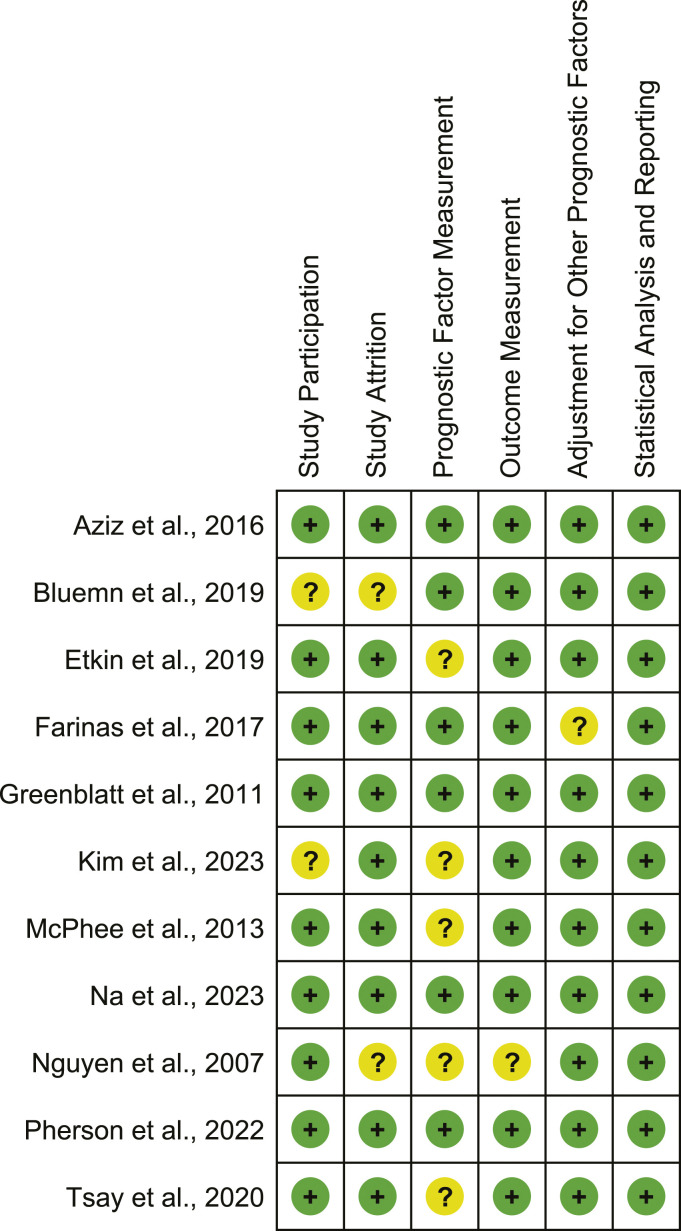
Risk of bias among the included studies according to the Quality in Prognosis Studies Tool.^25^ Green color and/or + indicates a low risk of bias and yellow and/or ? indicates a moderate risk of bias.

Study risk of bias is outlined in Figure 2. Two studies^7,11^ had a moderate risk of bias for study participation as the inclusion and exclusion criteria for selecting participants were inadequately explained. Two others^11,17^ had a moderate risk of bias for study attrition as the characteristics of participants lost to follow-up were inadequately described. Five studies^7,10,12,15,17^ had a moderate risk of bias for prognostic factor measurement as a clear SSI definition was not provided and/or because it was unclear whether the method used for SSI measurement was valid and reliable. One study^17^ had a moderate risk of bias for outcome measurement as a clear and clinically useful definition/description was not provided for all outcomes. One study^13^ had a moderate risk of bias for adjustment for other prognostic factors as it was unclear whether all potential confounders were accounted for in study design and analysis. Only four studies^8–10,16^ adjusted for our minimum confounder set.^39^ The median number of variables adjusted for in multivariable analyses across studies was 13 (IQR = 9–17).

### Primary outcome

**Figure 3.**
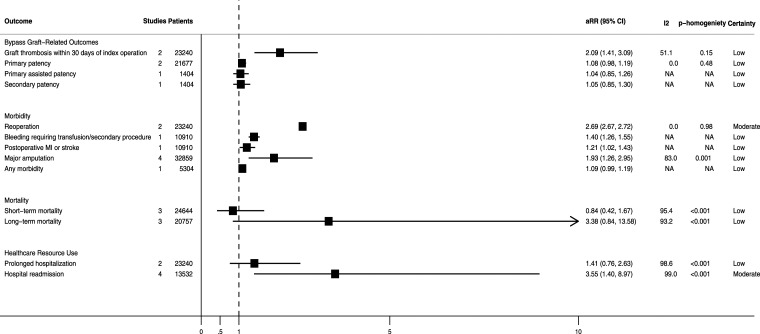
Pooled and unpooled adjusted outcomes associated with the development of surgical site infection after lower-limb revascularization surgery. aRR, adjusted relative risk; CI, confidence interval; MI, myocardial infarction; NA, not applicable.

Adjusted associations between development of an SSI and clinical outcomes and healthcare resource use after lower-limb revascularization surgery are summarized in Figure 3.

#### Bypass graft-related outcomes

Developing an SSI was associated with a significantly higher pooled adjusted-risk of bypass graft thrombosis within 30-days^8,16^ (aRR = 2.09; 95% CI = 1.41–3.09; n = 2 studies; n = 23,240 patients; I^2^ = 51.1%; low certainty). However, it was not associated with a significantly higher adjusted-risk of loss of primary^9,17^ (aRR = 1.08; 95% CI = 0.98–1.19; n = 2 studies; n = 21,677 patients; I^2^ = 0.0%; low certainty), primary-assisted^17^ (aRR = 1.04; 95% CI = 0.85–1.26; n = 1 study; n = 1404 patients; low certainty), or secondary^17^ (aRR = 1.05; 95% CI = 0.85–1.30; n = 1 study; n = 1404 patients; low certainty) bypass graft patency.

#### Morbidity

Developing an SSI was associated with an increased pooled adjusted-risk of reoperation^8,16^ (aRR = 2.69; 95% CI = 2.67–2.72; n = 2 studies; n = 23,240 patients; I^2^ = 0.0%; moderate certainty), bleeding requiring a transfusion or secondary procedure^8^ (aRR = 1.40; 95% CI = 1.26–1.55; n = 1 study; n = 10,910 patients; low certainty), myocardial infarction (MI) or stroke^8^ (aRR = 1.21; 95% CI = 1.02–1.43; n = 1 study; n = 10,910 patients; low certainty), and major (i.e., above-ankle) amputation^8,9,12,17^ (aRR = 1.93; 95% CI = 1.26–2.95; n = 4 studies; n = 32,859 patients; I^2^ = 83.0%; low certainty).

#### Mortality

Developing an SSI was not associated with significantly increased pooled adjusted-risk of short-term^8,16,17^ (defined as occurring ≤30-days following the index operation) (aRR = 0.84; 95% CI = 0.42–1.67; n = 3 studies; n = 24,644 patients; I^2^ = 95.4%; low certainty) or long-term^9,12,13^ (defined as occurring >30-days following the index operation) (aRR = 3.38; 95% CI = 0.84–13.58; n = 3 studies; n = 20,757 patients; I^2^ = 93.2%; low certainty) mortality.

#### Healthcare resource use

Developing an SSI was also not associated with a significantly increased pooled adjusted-risk of prolonged hospitalization^8,16^ (aRR = 1.41; 95% CI = 0.76–2.63; n = 2 studies; n = 23,240 patients; I^2^ = 98.6%; low certainty). However, the pooled adjusted-risk of hospital readmission^7,10,11,15^ was significantly higher in patients who developed an SSI (aRR = 3.55; 95% CI = 1.40–8.97; n = 4 studies; n = 13,532 patients; I^2^ = 99.0%; moderate certainty).

### Secondary outcomes

#### Associations between prosthetic graft SSI and outcomes

The adjusted-risk of major amputation^12^ (aRR = 6.72; 95% CI = 3.21–12.70; n = 1 study; n = 272 patients; low certainty) and long-term mortality^12^ (aRR = 6.40; 95% CI = 3.32–12.36; n = 1 study; n = 272 patients; low certainty) were significantly higher in patients who developed a prosthetic graft infection (Supplemental Figure 1).

#### Associations between early (≤30-days) and late (>30 days) SSI and outcomes

**Figure 4.**
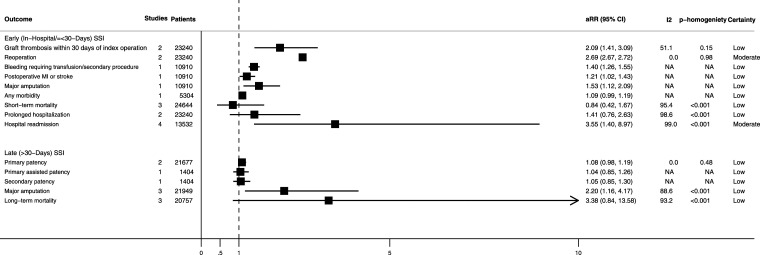
Pooled and unpooled adjusted outcomes associated with the development of early (in-hospital/≤30-days) and late (>30-days) surgical site infection after lower-limb revascularization surgery. aRR, adjusted relative risk; CI, confidence interval; MI, myocardial infarction; NA, not applicable.

The adjusted association between development of any of the predefined clinical or healthcare resource use outcomes did not differ significantly among those developing any SSI (i.e., an SSI occurring any time after the index operation) compared to an early (i.e., an SSI developing ≤30-days after the index operation) or late (i.e., an SSI developing >30-days after the index operation) SSI (Figure 4).

#### Sensitivity analysis, stratified meta-analyses, and meta-regression

Most sensitivity analyses yielded similar conclusions to the primary meta-analysis (Supplemental Table 3). The association between SSI and major amputation retained its significance when the study^12^ reporting on the association between prosthetic graft infection and amputation was removed from the main analysis (aRR = 1.54; 95% CI = 1.26–1.89; n = 3 studies; n = 32,587 patients; I^2^ = 28.8%; low certainty). However, although developing an SSI was not associated with an increased pooled adjusted-risk of prolonged hospitalization in the primary meta-analysis, it was in a sensitivity analysis that excluded one study^8^ in which the aOR was converted to an aRR^33^ (aRR = 1.93; 95% CI = 1.89–1.97; n = 1 study; n = 12,330 patients; low certainty). Further, while SSI was associated with a significantly increased pooled adjusted-risk of major amputation in the primary meta-analysis, it was not in a sensitivity analysis in which a larger study with an estimate that adjusted for fewer confounding factors^8^ was substituted for a smaller study^10^ with an estimate adjusted for more confounding factors (aRR = 1.71; 95% CI = 0.96–3.06; n = 4 studies; n = 27,253 patients; I^2^ = 87.9; low certainty). Stratified meta-analysis suggested a significantly higher pooled adjusted-risk of hospital readmission in studies that did not adjust for our minimum confounder set^39^ (*p* < 0.001) (Supplemental Table 4). Other stratified meta-analyses and meta-regressions were non-significant (*p* < 0.05 for all).

#### Publication bias

Egger’s test^43^ suggested evidence of small study effects potentially due to publication bias for the meta-analyses examining associations between development of an SSI and prolonged hospitalization (*p* < 0.001) and major amputation (*p* = 0.006). There was no evidence of small study effects for any of the other meta-analyses (*p* > 0.05 for all others).

## Discussion

This systematic review and meta-analysis identified 11 studies that enrolled a total of 61,628 patients and reported adjusted-associations between development of an SSI and 13 different outcomes after mostly infrainguinal bypass for CLTI. Our findings suggest that developing an SSI in this setting is associated with an increased adjusted-risk of hospital readmission, bypass graft thrombosis, reoperation, bleeding requiring a transfusion or secondary procedure, MI or stroke, and major amputation. However, while our finding of an increased pooled adjusted-risk of major amputation was somewhat sensitive to the degree of adjustment for confounding, it remained significant when the study^12^ examining the association between prosthetic graft infection and amputation was removed from the main meta-analysis. Developing an SSI >30-days after the index operation was associated with an adjusted-risk of major amputation. Prosthetic graft infection was associated with a significant and markedly increased adjusted-risk of major amputation and long-term mortality. Finally, stratified meta-analyses and meta-regression suggested that the association between SSI development and the adjusted-risk of hospital readmission was stronger in studies that did not adjust for our pre-specified minimum confounder set.^39^

Although the above mentioned associations are based on low-to-moderate certainty non-randomized evidence (which are often not causal), there are reasons to believe they may be causal. SSIs have been reported to represent the leading cause of unplanned and potentially preventable hospital readmissions after vascular surgery.^2,45^ Readmission is often required to manage infected wounds,^2,46^ which may require reoperation if the SSI extends into subcutaneous tissues or there is a concern for arterial involvement.^47^ Bleeding requiring a transfusion or secondary procedure after SSI development may result from anastomotic disruption or pseudoaneurysm formation and subsequent arterial hemorrhage.^48,49^ Development of an SSI may increase the risk of MI or stroke through a process termed immunothrombosis,^50,51^ whereby infection induces systemic inflammation, which in turn upregulates prothrombotic pathways.^52,53^ Infection-induced MI or stroke secondary to immunothrombosis has also been reported in patients with COVID-19,^54,55^ pneumococcal pneumonia,^56,57^ and influenza.^58,59^ Finally, development of an SSI may increase the risk of major amputation through inflammation-mediated disruption of the graft-artery anastomosis.^60,61^ Exposure of the graft to bacteria may also lead to bacterial attachment and biofilm formation,^62,63^ which may culminate in graft thrombosis and possibly distal thromboembolism.^48,64^ The significantly higher risk of late mortality in patients developing prosthetic graft infection may be secondary to the increased risk of major amputation as limb loss in patients with PAD has been reported to have an associated 1-year mortality risk as high as 50%.^65,66^

Our systematic review has limitations. First, the included studies used different classification systems (CDC,^26^ Szilagyi,^27^ etc.) to define and grade SSI. These differences may have led to exposure misclassification bias. However, the misclassification bias in this case would likely be non-differential and expected to bias our meta-analysis estimates towards the null (no associated difference), suggesting that our pooled outcome associations may be conservative.^67^ Second, the included studies used varying eligibility criteria, which may have introduced clinical heterogeneity into our meta-analyses.^68^ Further, many studies did not report important patient and procedural details (e.g., proportion of patients presenting with CLTI, undergoing urgent/emergent surgery, or receiving different lower-limb revascularization procedures such as patch angioplasties or infrainguinal prosthetic bypasses). Although the above could limit the external validity of our findings, it may also suggest that our reported outcomes more closely resemble the SSI-outcome associations observed in “real-world” practice.^69^ Third, because accounting for confounding is often felt to be critical in systematic reviews of prognostic studies,^19^ we only included studies reporting associations adjusted for potential confounders. We also performed several prespecified stratified meta-analyses and meta-regressions to determine whether our SSI-outcome associations varied based on whether studies adjusted for a prespecified minimum confounder set,^39^ above/equal to or below the median number of confounders used for adjustment, or based on the prevalence of important patient and procedural characteristics within included studies. Despite this, our findings may be limited by residual confounding.^70,71^ Finally, although we attempted to pool as many reported SSI-outcome associations as possible by transforming effect estimates reported using other measures of association (i.e., aORs and aHRs) to aRRs using validated methods^33,34^ (as has been done in other meta-analyses^35,36^), heterogeneity in outcome reporting limited the number of studies that could be included in our meta-analyses. In particular, most outcomes were only reported among participants developing either early or late SSI, preventing the direct comparison of whether the adjusted-risk for most of the identified clinical and healthcare resource use outcomes varied based on the timepoint at which participants developed an SSI.

This study has important implications for clinical practice and future research. Our findings highlight the value of using SSI as a quality metric after lower-limb revascularization surgery. Knowledge of our findings may motivate clinicians and healthcare systems to follow current best practices for SSI prevention and develop novel strategies intended to prevent and treat SSI.^72^ Our findings may also assist healthcare service managers in forecasting resource demands associated with these procedures.^7^ However, because our findings are based on low-to-moderate certainty evidence, they should be confirmed by large, multicenter, cohort studies whose design may be informed by the findings of this systematic review.^73^ Future cohort studies should ideally be prospective to minimize exposure and outcome misclassification bias.^67^ They should also use a clear and common SSI classification system, such as the CDC classification,^26^ to ensure that patients with similar exposure experience are recruited. Investigators should also consider establishing a clearly defined, clinically useful core outcomes set^74,75^ (including those identified as being associated with SSI in this systematic review and meta-analysis) and utilize a common system for diagnosing prosthetic graft infection, such as the Management of Aortic Graft Infection Collaboration (MAGIC) criteria, which has been suggested for use for peripheral bypass graft infections.^76^ They should also consider alternative approaches to outcome ascertainment and analyses that allow for incorporation of competing events or outcome severity such as time-to-event analyses^77^ or ordinal regression.^78^ Finally, investigators should adequately report and adjust their associations for patient and procedural characteristics known to be associated with both SSI and outcomes, including undergoing urgent/emergent surgery, receiving specific lower-limb revascularization procedures, or presenting with CLTI.^1,16,40–42,79,80^ Other patient and procedural factors that authors should strongly consider reporting and adjusting for within their multivariable analysis include those within our minimum confounder set^39^ along with any others felt supported by the literature.

## Conclusion

This systematic review and meta-analysis suggests that developing an SSI after lower-limb revascularization surgery is associated with an increased adjusted-risk of hospital readmission, bypass graft thrombosis, reoperation, bleeding requiring a transfusion or secondary procedure, MI or stroke, and major amputation. Prosthetic graft infection was also associated with a significant and markedly increased adjusted-risk of major amputation and long-term mortality. These findings suggest that SSI is a valuable quality metric and targeted SSI prevention and treatment strategies are needed for adults undergoing these procedures. However, as our estimates were based on heterogeneous, low-to-moderate certainty evidence, they should be confirmed by large, multicenter, cohort studies whose design may be informed by results of this systematic review.

## Supplemental Material

Supplemental Material - A systematic review and meta-analysis of outcomes associated with development of surgical site infection after lower-limb revascularization surgery Supplemental Material for A systematic review and meta-analysis of outcomes associated with development of surgical site infection after lower-limb revascularization surgery by Aidan M Kirkham, Jasmine Candeliere, Sudhir K Nagpal, Henry T Stelfox, Dalibor Kubelik, George Hajjar, Derek R MacFadden, Daniel I McIsaac, and Derek J Roberts in Vascular.
