# Supplementary material for: A systematic review and meta-analysis of outcomes associated with development of surgical site infection after lower-limb revascularization surgery
Source: Vascular. 2024 Oct 3;33(5):1150–61. doi: 10.1177/17085381241290039 (PMC12450249; doi:10.1177/17085381241290039)
Supplement: Supplemental Material - A systematic review and meta-analysis of outcomes associated with development of surgical site infection after lower-limb revascularization surgery [file sj-pdf-1-vas-10.1177_17085381241290039.pdf]

Supplemental Table 1. Preferred Reporting Items in Systematic Reviews and Meta-Analyses Checklist.

Section/topic	#	Checklist item	Reported on page #
**TITLE**			
Title	1	Identify the report as a systematic review, meta-analysis, or both.	Page 1
**ABSTRACT**			
Structured summary	2	Provide a structured summary including, as applicable: background; objectives; data sources; study eligibility criteria, participants, and interventions; study appraisal and synthesis methods; results; limitations; conclusions and implications of key findings; systematic review registration number.	Pages 4-5
**INTRODUCTION**			
Rationale	3	Describe the rationale for the review in the context of what is already known.	Page 6
Objectives	4	Provide an explicit statement of questions being addressed with reference to participants, interventions, comparisons, outcomes, and study design (PICOS).	Pages 6-7
**METHODS**			
Protocol and registration	5	Indicate if a review protocol exists, if and where it can be accessed (e.g., Web address), and, if available, provide registration information including registration number.	Page 7
Eligibility criteria	6	Specify study characteristics (e.g., PICOS, length of follow-up) and report characteristics (e.g., years considered, language, publication status) used as criteria for eligibility, giving rationale.	Pages 7-8
Information sources	7	Describe all information sources (e.g., databases with dates of coverage, contact with study authors to identify additional studies) in the search and date last searched.	Page 8
Search	8	Present full electronic search strategy for at least one database, including any limits used, such that it could be repeated.	Supplemental Table 2
Study selection	9	State the process for selecting studies (i.e., screening, eligibility, included in systematic review, and, if applicable, included in the meta-analysis).	Pages 8-9
Data collection process	10	Describe method of data extraction from reports (e.g., piloted forms, independently, in duplicate) and any processes for obtaining and confirming data from investigators.	Page 9

Data items	11	List and define all variables for which data were sought (e.g., PICOS, funding sources) and any assumptions and simplifications made.	Page 9
Risk of bias in individual studies	12	Describe methods used for assessing risk of bias of individual studies (including specification of whether this was done at the study or outcome level), and how this information is to be used in any data synthesis.	Page 9
Summary measures	13	State the principal summary measures (e.g., risk ratio, difference in means).	Page 9
Synthesis of results	14	Describe the methods of handling data and combining results of studies, if done, including measures of consistency (e.g., I^2) for each meta-analysis.	Pages 9-11

**Section/topic**	**#**	**Checklist item**	**Reported on page #**
Risk of bias across studies	15	Specify any assessment of risk of bias that may affect the cumulative evidence (e.g., publication bias, selective reporting within studies).	Page 12
Additional analyses	16	Describe methods of additional analyses (e.g., sensitivity or subgroup analyses, meta-regression), if done, indicating which were pre-specified.	Pages 11-12
**RESULTS**			
Study selection	17	Give numbers of studies screened, assessed for eligibility, and included in the review, with reasons for exclusions at each stage, ideally with a flow diagram.	Page 12, Figure 1
Study characteristics	18	For each study, present characteristics for which data were extracted (e.g., study size, PICOS, follow-up period) and provide the citations.	Page 13, Table 1
Risk of bias within studies	19	Present data on risk of bias of each study and, if available, any outcome level assessment (see item 12).	Pages 13-14, Figure 2
Results of individual studies	20	For all outcomes considered (benefits or harms), present, for each study: (a) simple summary data for each intervention group (b) effect estimates and confidence intervals, ideally with a forest plot.	Pages 14-16, Figures 3-4, Supplemental Figure 1

Synthesis of results	21	Present results of each meta-analysis done, including confidence intervals and measures of consistency.	Pages 14-16, Figures 3-4, Supplemental Figure 1
Risk of bias across studies	22	Present results of any assessment of risk of bias across studies (see Item 15).	Page 17
Additional analysis	23	Give results of additional analyses, if done (e.g., sensitivity or subgroup analyses, meta-regression [see Item 16]).	Pages 16-17, Supplemental Tables 3 and 4
**DISCUSSION**			
Summary of evidence	24	Summarize the main findings including the strength of evidence for each main outcome; consider their relevance to key groups (e.g., healthcare providers, users, and policy makers).	Pages 17-18
Limitations	25	Discuss limitations at study and outcome level (e.g., risk of bias), and at review-level (e.g., incomplete retrieval of identified research, reporting bias).	Page 18-19
Conclusions	26	Provide a general interpretation of the results in the context of other evidence, and implications for future research.	Pages 19-21
**FUNDING**			
Funding	27	Describe sources of funding for the systematic review and other support (e.g., supply of data); role of funders for the systematic review.	Pages 2, 22

From: Moher D, Liberati A, Tetzlaff J, Altman DG, The PRISMA Group (2009). Preferred Reporting Items for Systematic Reviews and Meta-Analyses: The PRISMA Statement. PLoS Med 6(7): e1000097. doi:10.1371/journal.pmed1000097

For more information, visit: **www.prisma-statement.org**.

Supplemental Table 2. Database Search Strategies (Inception to April 4th, 2023).

Search Theme	Search Terms			
	Ovid MEDLINE, PubMed, and Evidence-Based Medicine Reviews		Ovid EMBASE	
	Exploded MeSH Terms	Title and Subject Keywords	Exploded Emtree Terms	Title and Subject Keywords
Lower Limb Revascularization Surgery	Blood Vessel Prosthesis Implantation	aortobifemoral bypass* OR ((iliofemoral OR femoral OR femoral artery*) adj3 (endarterectom* OR patch* OR repair*)) OR ((femoral-distal OR femoral distal OR femoral-popliteal OR femoral popliteal OR femoral-tibial OR femoral tibial OR infrageniculate OR suprageniculate OR infrainguinal OR lower extremity OR lower limb OR peripheral vascular) adj3 (arterial surg* OR arterial bypass* OR bypass* OR bypass graft* OR bypass surg* OR graft* OR intervention* OR revascularization* OR revascularization procedure* OR vascular bypass* OR vascular	Artery Reconstruction, Aortofemoral Bypass, Endarterectomy	aortobifemoral bypass* OR ((iliofemoral OR femoral OR femoral artery*) adj3 (endarterectom* OR patch* OR repair*)) OR ((femoral-distal OR femoral distal OR femoral-popliteal OR femoral popliteal OR femoral-tibial OR femoral tibial OR infrageniculate OR suprageniculate OR infrainguinal OR lower extremity OR lower limb OR peripheral vascular) adj3 (arterial surg* OR arterial bypass* OR bypass* OR bypass graft* OR bypass surg* OR graft* OR intervention* OR revascularization* OR revascularization procedure* OR vascular bypass* OR vascular

		bypass surg* OR vascular graft* OR vein graft* OR prosthetic graft*))		bypass surg* OR vascular graft* OR vein graft* OR prosthetic graft*))
Infection	Surgical Wound Infection	infection* OR surgical site infection* OR wound infection*	Surgical Infection	infection* OR surgical site infection* OR wound infection*

Where MeSH, Medical Subject Heading.

Supplemental Figure 1. Unpooled Adjusted Outcomes Associated with Development of Prosthetic Graft Surgical Site Infection After Lower Limb Revascularization Surgery.

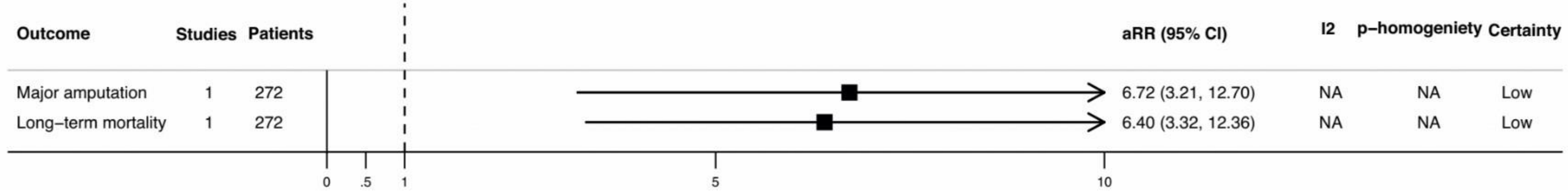

Where aRR, Adjusted Relative Risk; CI, Confidence Interval; NA, Not Applicable.

Supplemental Table 3. Sensitivity Analysis Examining Clinical Outcomes Associated with Any Surgical Site Infection Following Lower Limb Revascularization Surgery.

**Outcome**	**No.**		**aRR (95% CI)**	**Heterogeneity in Pooled Estimate**		**Egger's Test (p-value)**	**Certainty**
	**Studies**	**Patients**		**I^2 (%)**	**p-homogeneity**		
**Healthcare Resource Use**							
Prolonged hospitalization (excluding Na *et al.*, 2023[8])	1	12330	1.93 (1.89-1.97)	NA	NA	NA	Low
Hospital readmission (including Aziz *et al.*, 2016[14] vs Tsay *et al.*, 2020[10])	4	10874	5.04 (4.02-6.30)	78.8	**0.003**	0.12	Moderate
**Graft-Related Outcomes**							
Bypass graft thrombosis (excluding Na *et al.*, 2023[8])	3	34007	1.41 (0.76-2.62)	99.8	**<0.001**	0.081	Low
**Morbidity**							
Major amputation (including Tsay *et al.*,[10] 2022 vs Na *et al.*, 2023[8])	4	27253	1.71 (0.96-3.06)	87.9	**<0.001**	0.62	Low
Major amputation (excluding Etkin *et al.*, 2019[12])	3	32587	1.54 (1.26-1.89)	28.8	0.78	0.12	Low
Reoperation (excluding Na *et al.*, 2023[8])	1	12330	2.69 (2.67-2.72)	NA	NA	NA	Moderate
**Short- and Long-Term Mortality**							
Short-term mortality (including Tsay *et al.*, 2020[10] vs Na *et al.*,	3	19038	0.98 (0.51-1.91)	96.3	**<0.001**	0.24	Low

2023[8])							
Long-term mortality (excluding Etkin *et al*., 2019[12])	2	20485	2.40 (0.45-12.88)	86.5	**0.007**	**0.006**	Low

Where aRR, Adjusted Relative Risk; CI, Confidence Interval and NA, Not Applicable.

Supplemental Table 4. Stratified Meta-Analysis and Meta-Regression Examining Clinical Outcomes Associated with Any Surgical Site Infection Following Lower Limb Revascularization Surgery According to Study Risk of Bias Ratings for the Six Domains Within

the Quality in Prognosis Studies Tool,[25] Whether or Not They Adjusted for The Prespecified Minimum Confounder Set[39] and Whether They Adjusted Above/Equal to or Below the Median Number of Confounders (n=13).

STRATIFIED META-ANALYSES								
**Outcome**	**Low (or adjusted for above/equal to median number of confounders; or adjusted for minimum confounder set*)**			**Moderate/High (or adjusted for below median number of confounders; or did not adjust for or did not adjust for minimum confounder set*)**			**p-value between groups**	**p-value from meta-regression**
	**No. studies**	**aRR (95 % CI)**	**I^2 (%) (p-homogeneity)**	**No. studies**	**aRR (95% CI)**	**I^2 (%) (p-homogeneity)**		
**Study Participation**								
Major amputation	4	1.93 (1.26-2.95)	83.0 (**0.001**)	NA	NA	NA	NA	NA
Short-term mortality	3	0.84 (0.42-1.67)	95.4 (**<0.001**)	NA	NA	NA	NA	NA
Long-term mortality	3	3.38 (0.84-13.58)	93.2 (**<0.001**)	NA	NA	NA	NA	NA
Hospital readmission	2	2.28 (0.57-9.10)	98.6 (**<0.001**)	2	5.59 (4.09-7.65)	81.9 (**0.019**)	0.22	0.34
**Study Attrition**								
Bypass graft thrombosis	3	1.59 (0.87-2.91)	97.8 (**<0.001**)	1	1.05 (0.92-1.19)	NA	0.18	0.46
Major amputation	3	2.24 (1.18-4.25)	88.4 (**<0.001**)	1	1.47 (1.09-1.99)	NA	0.25	0.68
Short-term mortality	2	0.59 (0.56-0.62)	0.0 (0.76)	1	1.40 (1.09-1.81)	NA	**<0.001**	0.096
Long-term mortality	3	3.38 (0.84-13.58)	93.2 (**<0.001**)	NA	NA	NA	NA	NA
Hospital readmission	3	2.90 (1.01-8.28)	98.5 (**<0.001**)	1	6.47 (5.68-7.37)	NA	0.14	0.46
**Prognostic Factor Measurement**								
Bypass graft	3	1.59 (0.87-2.91)	97.8 (**<0.001**)	1	1.05 (0.92-1.19)	NA	0.18	0.46

thrombosis								
Major amputation	2	1.48 (1.21-1.82)	0.0 (0.77)	2	3.05 (0.69-13.46)	93.6 (<**0.001**)	0.35	0.45
Short-term mortality	2	0.59 (0.56-0.62)	0.0 (0.76)	1	1.40 (1.09-1.81)	NA	**<0.001**	0.096
Long term mortality	2	2.40 (0.45-12.88)	86.5 (<**0.001**)	1	6.40 (3.32-12.36)	NA	0.29	0.62
Hospital readmission	1	6.47 (5.68-7.37)	NA	3	2.90 (1.01-8.28)	98.5 (<**0.001**)	0.14	0.46
**Outcome Measurement**								
Bypass graft thrombosis	3	1.59 (0.87-2.91)	97.8 (<**0.001**)	1	1.05 (0.92-1.19)	NA	0.18	0.46
Major amputation	3	2.24 (1.18-4.25)	88.4 (<**0.001**)	1	1.47 (1.09-1.99)	NA	0.25	0.68
Short-term mortality	2	0.59 (0.56-0.62)	0.0 (0.76)	1	1.40 (1.09-1.81)	NA	**<0.001**	0.096
Long-term mortality	3	3.38 (0.84-13.58)	93.2 (<**0.001**)	NA	NA	NA	NA	NA
Hospital readmission	4	3.55 (1.40-8.97)	99.0 (<**0.001**)	NA	NA	NA	NA	NA
**Adjustment for Other Prognostic Factors**								
Major amputation	4	1.93 (1.26-2.95)	83.0 (**0.001**)	NA	NA	NA	NA	NA
Short-term mortality	3	0.84 (0.42-1.67)	95.4 (<**0.001**)	NA	NA	NA	NA	NA
Long-term mortality	2	2.62 (0.48-14.29)	95.8 (<**0.001**)	1	6.35 (1.87-21.55)	NA	0.41	0.68
Hospital readmission	4	3.55 (1.40-8.97)	99.0 (<**0.001**)	NA	NA	NA	NA	NA
**Statistical Analysis and Reporting**								
Major amputation	4	1.93 (1.26-2.95)	83.0 (**0.001**)	NA	NA	NA	NA	NA
Short-term	3	0.84 (0.42-1.67)	95.4 (<**0.001**)	NA	NA	NA	NA	NA

mortality								
Long-term mortality	3	3.38 (0.84-13.58)	93.2 (<**0.001**)	NA	NA	NA	NA	NA
Hospital readmission	4	3.55 (1.40-8.97)	99.0 (<**0.001**)	NA	NA	NA	NA	NA
**Adjusted for Minimum Confounder Set***								
Bypass graft thrombosis	3	1.59 (0.87-2.91)	97.8 (<**0.001**)	1	1.05 (0.92-1.19)	NA	0.18	0.46
Major amputation	2	1.48 (1.21-1.82)	0.0 (0.77)	2	3.05 (0.69-13.46)	93.6 (<**0.001**)	0.35	0.45
Short-term mortality	2	0.59 (0.56-0.62)	0.0 (0.76)	1	1.40 (1.09-1.81)	NA	**<0.001**	0.096
Long-term mortality	1	1.13 (0.89-1.44)	NA	2	6.39 (3.58-11.40)	0.0 (0.99)	<**0.001**	0.12
Hospital readmission	1	1.13 (0.97-1.32)	NA	3	5.32 (4.16-6.81)	75.4 (**0.017**)	**<0.001**	**0.020†**
**Adjusted Above/Equal to or Below the Median Number of Confounders (n=13)**								
Bypass graft thrombosis	3	1.59 (0.87-2.91)	97.8 (<**0.001**)	1	1.05 (0.92-1.19)	NA	0.18	0.46
Major amputation	2	1.48 (1.21-1.82)	0.0 (0.77)	2	3.05 (0.69-13.46)	93.6 (<**0.001**)	0.35	0.45
Short-term mortality	2	0.59 (0.56-0.62)	0.0 (0.76)	1	1.40 (1.09-1.81)	NA	**<0.001**	0.096
Long-term mortality	1	1.13 (0.89-1.44)	NA	2	6.39 (3.58-11.40)	0.0 (0.99)	<**0.001**	0.12
Hospital readmission	2	2.28 (0.57-9.10)	98.6 (<**0.001**)	2	5.59 (4.09-7.65)	81.9 (**0.019**)	0.22	0.34
**META-REGRESSION ANALYSES**								
**Patient or Procedural Characteristic**		**Number of Studies**		**Regression Coefficient (95% CI)**		**SE**		**p-value**
**Major Amputation**								
CLTI		4		-2.48 (-6.83-1.87)		1.01		0.13
**Hospital Readmission**								

CLTI	4	-2.44 (-15.65-10.70)	3.07	0.51

Where aRR, Adjusted Relative Risk; CI, Confidence Interval; CLTI, Chronic Limb Threatening Ischemia; NA, Not Applicable and SE, Standard Error.

*Minimum confounder set[39] includes age, sex, obesity, diabetes, chronic limb threatening ischemia and groin incision.

†Indicates statistical significance.
